# Tactile Gap Detection Deteriorates during Bimanual Symmetrical Movements under Mirror Visual Feedback

**DOI:** 10.1371/journal.pone.0146077

**Published:** 2016-01-05

**Authors:** Janet H. Bultitude, Georgiana Juravle, Charles Spence

**Affiliations:** 1 Department of Psychology, University of Bath, Bath, North East Somerset, United Kingdom; 2 The Centre for Functional Magnetic Resonance Imaging of the Brain, University of Oxford, Oxford, Oxfordshire, United Kingdom; 3 Department of Systems Neuroscience, University Medical Center Hamburg-Eppendorf, Hamburg, Germany; 4 Crossmodal Research Laboratory, Department of Experimental Psychology, Oxford University, Oxford, Oxfordshire, United Kingdom; Duke University, UNITED STATES

## Abstract

It has been suggested that incongruence between signals for motor intention and sensory input can cause pain and other sensory abnormalities. This claim is supported by reports that moving in an environment of induced sensorimotor conflict leads to elevated pain and sensory symptoms in those with certain painful conditions. Similar procedures can lead to reports of anomalous sensations in healthy volunteers too. In the present study, we used mirror visual feedback to investigate the effects of sensorimotor incongruence on responses to stimuli that arise from sources external to the body, in particular, touch. Incongruence between the sensory and motor signals for the right arm was manipulated by having the participants make symmetrical or asymmetrical movements while watching a reflection of their left arm in a parasagittal mirror, or the left hand surface of a similarly positioned opaque board. In contrast to our prediction, sensitivity to the presence of gaps in tactile stimulation of the right forearm was not reduced when participants made asymmetrical movements during mirror visual feedback, as compared to when they made symmetrical or asymmetrical movements with no visual feedback. Instead, sensitivity was reduced when participants made symmetrical movements during mirror visual feedback relative to the other three conditions. We suggest that small discrepancies between sensory and motor information, as they occur during mirror visual feedback with symmetrical movements, can impair tactile processing. In contrast, asymmetrical movements with mirror visual feedback may not impact tactile processing because the larger discrepancies between sensory and motor information may prevent the integration of these sources of information. These results contrast with previous reports of anomalous sensations during exposure to both low and high sensorimotor conflict, but are nevertheless in agreement with a forward model interpretation of perceptual modulations during goal directed movement.

## Introduction

For any self-generated movement, the central nervous system produces an internal copy of the motor command that is cross-referenced against sensory input. This “efference copy” signals motor intention and allows us to differentiate between self-generated movements and those that are caused by external forces acting on our body [1]. Problems in generating or recognising efference copy are thought to contribute to symptoms such as auditory hallucinations in schizophrenia [2,3] and denial of hemiplegia in stroke patients [1]. Other pathologies may arise when the efference copy is recognised, but contradicts sensory signals. For example, Harris [4] suggested that incongruence between cortical signalling of motor intention and sensory input may lead to experiences of pain. This “sensorimotor” theory of pain may explain conditions in which pain and other symptoms arise in the absence of, or that are disproportionate to, any observable limb pathology, such as repetitive strain injury, musician’s dystonia, phantom limb pain, and Complex Regional Pain Syndrome (CRPS) [4,5]. Studies of those suffering from such conditions have revealed abnormal sensory processing [6–9] and changes in cortical representations of the affected limb [10–12]. These findings are consistent with Harris’ [4] suggestion that inaccurate sensory and motor signals stemming from altered receptive fields may aggravate the nociceptive system.

One way in which to investigate the effects of sensorimotor incongruence is by using mirror visual feedback. If a mirror is placed perpendicular to the torso and a person places their arms on either side, the hand in the mirror’s reflection will appear to occupy the same space as the hand behind the mirror. Performing bilateral symmetrical arm movements while watching the reflected image will result in the person having the impression that they are watching the hand positioned behind the mirror rather than the reflected image of the other hand. Incongruence between visual information and proprioceptive and motor signals can be created by having the participant watch the reflected image while making asymmetrical movements: The direction in which the arm appears to move will be opposite to the intended direction of movement and the direction in which the arm is felt to move. Several studies have examined the effects of sensorimotor incongruence by comparing reported limb sensations for participants performing symmetrical and asymmetrical movements during mirror visual feedback and control conditions in which the mirror is removed or replaced by an opaque board [13–18].

In support of the sensorimotor theory of pain, inducing sensorimotor incongruence using movements with mirror visual feedback has been found to *exacerbate* pain in those individuals with fibromyalgia [18] and whiplash [15], as well as in professional violinists with baseline sensory abnormalities ("violinist's dystonia" **[**17]**)**. In these studies, sensorimotor incongruence was also associated with increased self-reports of anomalous sensations in the limb, such as feelings of foreignness, tingling, and changes in perceived weight or temperature. Other studies have shown that healthy, pain-free volunteers exposed to sensorimotor incongruence also reported anomalous sensations, albeit with a lower frequency and severity than in the clinical populations [13,16,18]. However, unlike the participants with baseline pathology, healthy volunteers rarely reported pain during sensorimotor incongruence (see McCabe et al. [13]). Furthermore, sensorimotor incongruence did not alter pain thresholds in healthy volunteers [14]. Overall, it would appear that sensorimotor incongruence exacerbates pain in those individuals who already demonstrate baseline sensory abnormalities (which may include pain), but does not generally cause pain in asymptomatic individuals. In contrast, sensorimotor incongruence leads to anomalous sensations other than pain in both symptomatic and asymptomatic individuals.

McCabe and her colleagues [13] suggested that anomalous sensations during incongruent sensorimotor feedback might be treated as warning signals to alert the nervous system of potential errors in information processing, and that pain only results when the errors are particularly salient or persistent. Consistent with this view, anomalous symptoms during sensorimotor incongruence are reported less frequently in healthy volunteers than in those with baseline symptoms, for whom the information processing error is presumably greater and poses more of a threat [17,18]. Furthermore, there is some suggestion that the frequency and intensity of the reported sensory changes are greatest under conditions of maximum sensorimotor incongruence [16–19], although statistically significant differences between different degrees of incongruence have not always been obtained [13]. Overall, these studies suggest that sensorimotor incongruence can lead to abnormal bodily sensations.

What has not been assessed is whether sensorimotor incongruence can also lead to changes in responses to non-painful stimulation from sources external to the body. Reduced sensitivity to touch has been reported in patients with CRPS [6,7], violinist’s dystonia [20–22], and fibromyalgia [23]. It is possible that sensorimotor incongruence may also play a role in the manifestation of this sensory change. Reduced sensitivity to external stimulation could be driven by a reduction in the perceived reliability of sensory signals in the face of sensorimotor incongruence. As a first endeavour of this kind, the present study therefore investigated the effects of sensorimotor incongruence on the performance of healthy volunteers on a test of tactile sensitivity: the ability to detect a “gap” or pause in a sustained vibrotactile stimulus. That is, the task used here differed from previous studies in that the participants were required, for the first time, to perform a perceptual task. If the anomalous sensations and reduced tactile sensitivity that have been reported in various patient populations arise due to sensorimotor incongruence, then the tactile gap detection performed by our healthy participants should also be significantly affected by the experimental induction of sensorimotor incongruence. The prediction was that sensorimotor incongruence mediated by the manipulation of visual input would lead to lower sensitivity in the gap detection test as compared to those conditions in which no visual information was given about the limb. We expected that gap detection performance would be worse in the condition that induced the strongest sensorimotor incongruence–that is, when participants directed their gaze to the reflected image of their left hand while moving their hands in opposite directions.

In previous studies, reports of anomalous symptoms arose after mirror visual feedback with both symmetrical and asymmetrical movements (e.g., [13,16]). It has been assumed that these two conditions represent conditions of low and high sensorimotor incongruence, respectively. This assumption requires that in both conditions participants experience the illusion that they are viewing the hand that occupies the space behind the mirror rather than the reflected image of their other hand, and, furthermore, that the strength of this illusion is somewhat similar across the two movement conditions. It is, however, possible that there is inter-subject variability in the extent to which participants experience this illusion. For example, variability has been reported in the susceptibility of different individuals to another sensorimotor illusion: the rubber hand illusion (RHI, [24,25]). If some individuals experience the mirror visual feedback illusion only weakly, this would interfere with the induction of sensorimotor incongruence. In order to gain an understanding of whether there is such inter-individual variability in susceptibility to mirror visual feedback we therefore had our participants report the subjective strength of the illusion for both the symmetrical and asymmetrical movement conditions.

## Methods

### Participants

Twenty-three participants (mean age of 26 years; age range: 20–40; 6 males) took part. All of the participants were in good health and without pain and reported normal touch, movement, and hearing, as well normal or corrected-to-normal vision. Participants were right-handed according to self-report. The session lasted for approximately 60 minutes and the participants received £10 in return for taking part.

### Apparatus

A 45 x 30 cm mirror was positioned in the middle of the table such that it was in line with the participant’s mid-sagittal plane with the reflective surface facing left. A 45 x 30cm board was attached to the top edge of the mirror to prevent the participant from seeing over the top of the mirror when they looked into the reflective side. Another board (45 x 45cm) was positioned in front of the reflective surface of the mirror during those conditions in which the participants were not provided with vision of the reflection of their left arm.

The tactile stimulation protocol was adapted from a previous study in which participants made judgments about tactile stimuli that were applied to their forearm while they caught or threw a basketball [26]. The participants had one piezo actuator [VBW32 skin stimulator, 1.6 x 2.4 cm vibrating surface, Audiological Engineering Corp., Somerville, MA, USA] attached to the ventral surface of their right wrist with a wide elasticated Velcro strap. The tactile stimulator was wrapped in several layers of thin sponge in order to reduce the possibility that the participants would hear the operation of the stimulator. Following pilot testing, additional foam was positioned between the stimulator and the person’s wrist to reduce the possibility that they would be able to hear the operation of the stimulator through bone conduction and to decrease the overall intensity of the tactile stimulation. Two more straps secured the stimulator cable to the participant’s arm above and below the elbow so that it would not impede the movement of the arm. The stimulator was driven by means of a custom-built stimulator box that interfaced via the serial port with a Dell computer (Dell Technologies) using Matlab software (Psychophysics Toolbox 3; [27]). Vibration on the wrist consisted of two 250Hz, 12 dB sensation level pulses, that were either separated in time (GAP trials) or were consecutive such as to deliver a single, continuous, vibration (NO GAP trials). The vibratory stimulus was clearly above the perceptual detection threshold, as previously tested in studies from our lab [26,28]. The auditory signals were delivered via headphones (Phillips SBC HC060). White noise was presented through the same headphones for the duration of the experiment as an additional measure to prevent the participants from hearing the operation of the tactile stimulator. The participants gave vocal responses for each experimental trial that the experimenter entered into the computer.

### Procedure

The study protocol was approved by the University of Oxford Medical Sciences Inter-Divisional Research Ethics Committee (reference number MSD-IDREC-C1-2013-126) and written consent was obtained from all participants. Before the experiment began, participants were instructed in the different movement types. Participants first practiced making up and down movements of their hands and forearms in time with a metronome set to 1Hz. The locus of movement was restricted to the elbow joint. The fingers, hands, and forearms remained in a straight line relative to each other. The highest extent of the movement was level with the height of the top edge of the mirror and the lowest extent was approximately 5cm above the surface of the table. Participants were instructed to keep their forearms and hands clear of the table surface for the duration of each trial because contact with the table might cause variations in the intensity of tactile stimulation during the experiment. Once the participants became fluent in the movement the metronome was switched off and they continued practicing until the experimenter was satisfied that they had achieved a consistent speed and amplitude of movement. The metronome was not used during the experiment proper in order to prevent any potential conflict with the auditory cues.

**Fig 1:**
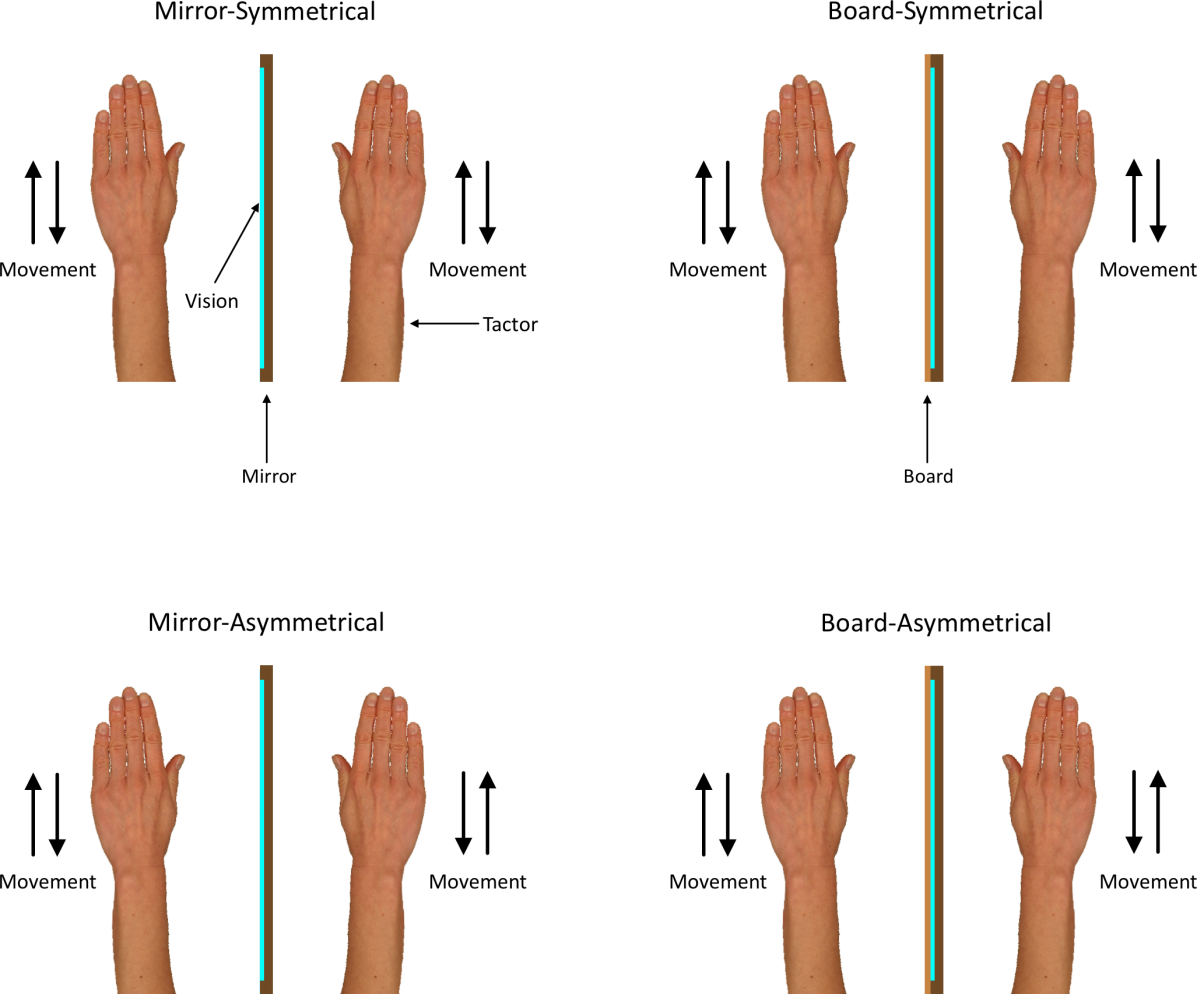
Representation of the four experimental conditions. The direction of vision and the position of the tactile stimulator were the same for all conditions.

The experiment consisted of two parts. First, the participants completed a pre-test to determine which gap length to use for the main experimental tasks. Second, after a break of approximately 5 minutes, the participants completed the main experiment, which consisted of four blocks corresponding to the four conditions: *mirror-symmetrical*, *mirror-asymmetrical*, *board-symmetrical*, and *board-asymmetrical* (as outlined in Fig 1).

At the beginning of the pre-test, the board was placed in front of the mirror and the participant positioned their arms on either side with their palms facing downward (see Fig 1). The participant angled their head to the left of their body midline such that they could look at the left surface of the board and their right hand and arm were occluded from view. The gap detection task was adapted from Juravle and Spence [28]. Each trial began with an auditory beep (400Hz, 50ms) signalling that the participant should start moving. The participant moved their hands repeatedly up and down in a symmetrical fashion at a rate of approximately 1Hz while looking at the centre of the board. The tactile stimulator started vibrating 1000 ms after the beep and lasted for 3,500ms. After a further 1000ms, a second beep (400Hz, 50ms) signalled the end of the trial. Participants returned their hands to the starting position and indicated to the experimenter whether or not they had felt a gap in the stimulation.

The procedure for the *board-symmetrical* block of the main experiment was identical to the pre-test. The procedure for the three other blocks of the main experimental blocks were similar to that for the pre-test and the *board-symmetrical* block with two exceptions: 1) In the *mirror-asymmetrical* and *board-asymmetrical* conditions, the participants moved their hands in opposite directions such that one hand reached the apex of the movement (level with the top of the mirror) at the same time as the other hand reached the base of the movement (approximately 5cm above the surface of the table); 2) During the *mirror-symmetrical* and *mirror-asymmetrical* conditions the board was removed thus revealing the reflective surface of the mirror. The participants were instructed to look at the reflection of their left hand in the mirror for the duration of the block and to “try to believe” that they were actually looking at their right hand. At the end of each of the *mirror-symmetrical* and *mirror-asymmetrical* blocks of trials, the strength of the visual illusion was measured by asking participants to rate the extent to which they felt that they were watching their right hand rather than the reflection of their left hand in the mirror on a scale of 0 (not at all) to 10 (completely).

### Design

The pre-test consisted of 10 repetitions for each of 8 gap durations [0(no gap), 10, 20, 30, 40, 50, 60, or 70 ms; adapted from 26]. The timing of the gap relative to the onset of the vibration varied randomly from between 1,000 to 2,500ms. The number of trials in which the participant stated there was a gap were counted for each condition and fitted with a cumulative Gaussian using a maximum-likelihood criterion. For each participant, the gap duration at which they detected the gap on 50% of trials was used as the gap duration for the main experimental blocks.

The *mirror-symmetrical*, *mirror-asymmetrical*, *board-symmetrical*, and *board-asymmetrical* blocks each consisted of 30 trials. Half of the trials were GAP trials (the vibration was interrupted for the length of time determined in the pre-test block) and half of the trials were NO GAP trials (the vibration was continuous), presented in a pseudorandom order. For each of the GAP trials, the timing of the gap relative to the onset of the vibration varied randomly from 1,000 to 2,500ms. The order in which the four blocks were presented was counterbalanced across participants.

### Preparation of Gap Detection Data

For each participant, the percentages of true positives (i.e., YES responses to a GAP trial) and false positives (i.e., YES responses to a NO GAP trial) were calculated separately for each of the four conditions. Sensitivity (d’) and criterion (c) were derived from these percentages using signal detection theory [29]. In those cases where all of the gaps were detected or else no false positives were registered, the proportions of 1 and 0 were adjusted by 1/(2N) and 1/(1–2N), respectively, where N is the number of trials [29].

## Results

The median rating of illusion strength during the *mirror-symmetrical* condition (Median = 7, range = 1–9) was significantly higher than during the *mirror-asymmetrical* condition [Median = 5, range = 1–8; *Z* = 4.03, *p*<0.001]. The median gap length was 30 (range = 11–80).

**Fig 2:**
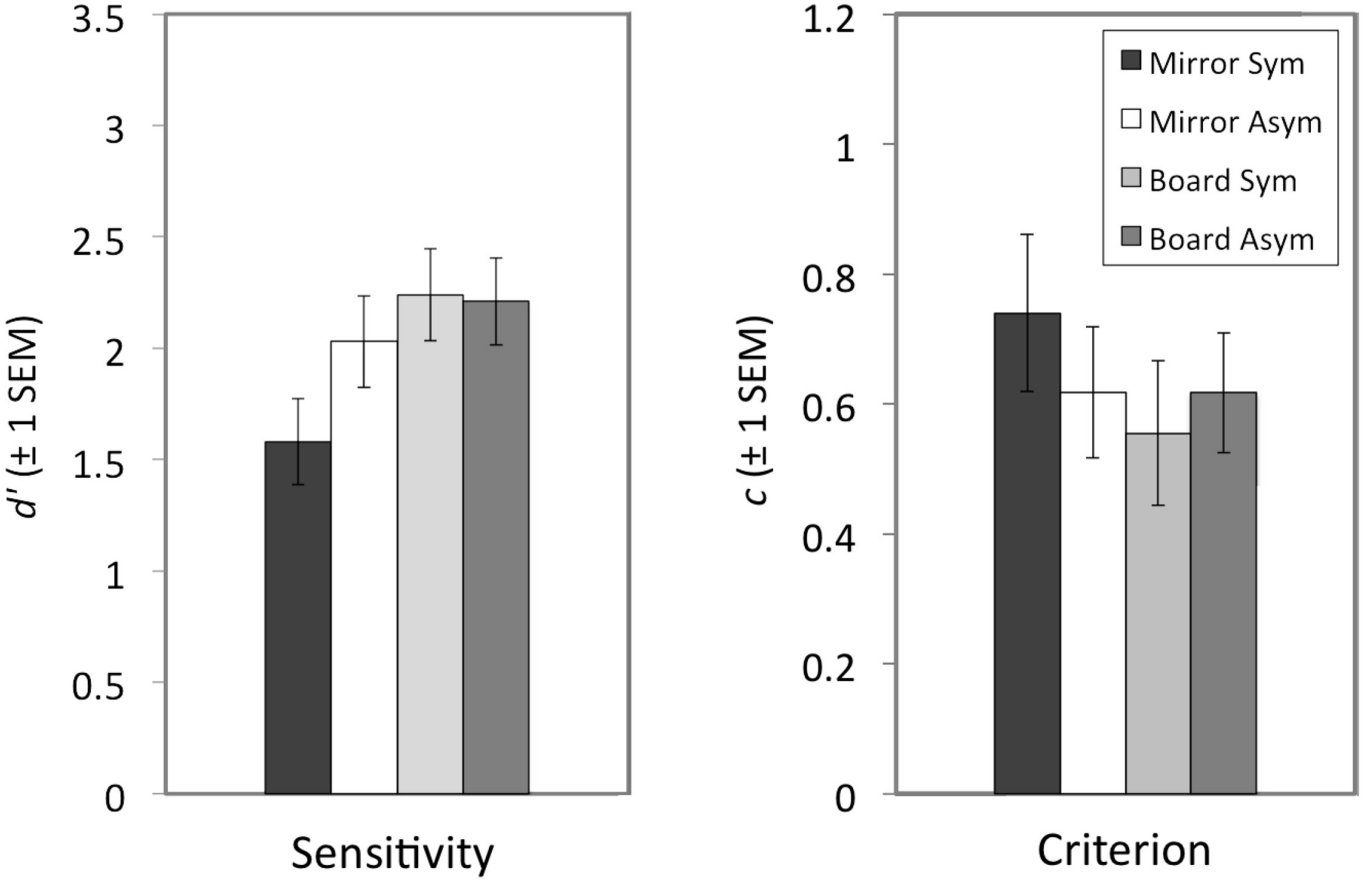
Mirror visual feedback during symmetrical, but not asymmetrical, arm movements impairs tactile gap detection. Sensitivity *d’* data are presented on the left and criterion *c* data are presented on the right. Error bars represent ±1 SEM.

The sensitivity and criterion data are provided in S1 Table and are summarised in Table 1 and Fig 2. The data were parametric and satisfied all assumptions of repeated-measures Analysis of Variance (ANOVA). The sensitivity data and criterion data were subjected to separate repeated-measures ANOVAs, with Mirror Condition (mirror, board) and Movement (symmetrical, asymmetrical) as within-participants factors. Significant interactions were further explored using paired t-tests with corrections for multiple comparisons using Holm’s sequential Bonferroni adjustment [30].

**Table 1:** Mean sensitivity and criterion data across the four experimental conditions.

	Mirror-Symmetrical	Mirror-Asymmetrical	Board-Symmetrical	Board-Asymmetrical
**Sensitivity (*d’*)**	1.58 (0.19)	2.03 (0.21)	2.24 (0.21)	2.21 (0.20)
**Criterion (*c*)**	0.74 (0.12)	0.62 (0.10)	0.56 (0.11)	0.62 (0.09)

Standard Errors of the Mean (SEMs) are indicated in brackets.

### Sensitivity (*d’*)

The repeated measures ANOVA showed a main effect of Mirror Condition [*F*(1,22) = 18.31, *p*<0.005, *η*_*p*_^*2*^ = 0.45], with lower sensitivity for the mirror conditions (*M* = 1.80, *SEM* = 0.19) than for the board conditions (*M* = 2.22, *SEM* = 0.19). There was also a significant main effect of Movement [*F*(1,22) = 5.72, *p*<0.05, *η*_*p*_^*2*^ = 0.21], with lower sensitivity in the symmetrical condition (*M* = 1.91, *SEM* = 0.19) than in the asymmetrical condition (*M* = 2.12, *p*<0.19). Finally, there was a significant Mirror Condition x Movement interaction [*F*(1,22) = 6.27, *p*<0.05, *η*_*p*_^*2*^ = 0.22]. Follow-up t-tests revealed that sensitivity was significantly lower for the *mirror-symmetrical* condition (*M* = 1.58, *SEM* = 0.19) than for the *mirror-asymmetrical* condition (*M* = 2.03, *SEM* = 0.21; *t*(22) = 3.53, *p*<0.005), the *board-symmetrical* condition (*M* = 2.24, *SEM* = 0.21; *t*(22) = 4.59, *p*<0.001), and the *board-asymmetrical* condition (*M* = 2.21, *SEM* = 0.20; *t*(22) = 4.92, *p*<001). None of the other contrasts were significant (*t*s<1.5, *p*s>0.14).

### Criterion (*c*)

There were no significant main effects and no significant interaction in the analysis of the criterion data (*F*s<3.80, *p*s>0.064).

## Discussion

The results show that manipulating visual information about a limb using mirror visual feedback reduces healthy participants’ tactile gap sensitivity relative to those conditions in which there was no visual feedback of the limb moving. That said, the direction of this effect appeared to be different than expected.

Drawing from the sensorimotor theory of pain, we predicted that compared to the three other conditions, gap detection performance would be worst when participants directed their gaze toward the reflected image of their left hand while moving their hands in opposite directions. This prediction arose particularly since this condition should induce the greatest degree of sensorimotor incongruence. Contrary to our prediction, however, the sensitivity of our participants’ judgments when they made asymmetrical movements with mirror visual feedback was no different to that for the two conditions in which there was no visual feedback of the limb. Instead, when participants made *symmetrical* movements with mirror visual feedback their sensitivity on the gap detection task was significantly lower than in all of the other conditions.

Tactile perception is degraded while our limbs are in motion, a phenomenon known as sensory suppression (e.g., [28,31,32]). It is likely that this degradation occurs because the incoming sensory stream is attenuated to cancel out self-generated sensations. This process is thought to involve the prediction of the sensory consequences of the movement, based on the efference copy, using an internal representation of the environment, or forward model [33–35]. Since all four experimental conditions in the current study involved tactile stimulation of a moving limb, the reduction in gap detection performance during symmetrical movements with mirror visual feedback must reflect an additional degree of sensory suppression. One possibility is that the small degree of sensorimotor incongruence that arises while making symmetrical movements during mirror visual feedback is sufficient for the forward model to generate larger estimates of self-generated sensations relative to the other conditions tested in this experiment, and therefore a greater degree of sensory suppression. We infer that for those other conditions tested here where a large discrepancy between the movement and visual feedback was available the forward model highlighted or emphasized the quality of tactile information with the direct result of a reduction in the predicted movement-related sensory suppression.

Such subtle incongruence could arise due to minor differences in the distance of each limb from the mirror or imperfect coordination of movements. Previous research suggests that although vision of the site of touch enhances tactile sensitivity when the seen and felt position of the touched site are in close alignment [36–39], misaligning visual and proprioceptive information about a hand by just a few centimetres can decrease sensitivity. The RHI, which can shift the felt position of the participant’s hand away from its true location and towards the location of a similarly oriented rubber hand [40], reduced attention to tactile stimulation of the manipulated hand relative to the participant’s other hand [41]. Similarly, in two separate experiments, Folegatti and his colleagues [42] documented reduced RTs to tactile stimulation when they used the RHI and prismatic displacement of vision to misalign the visual and proprioceptive representations of the hand. Finally, Tamé and his colleagues [39] demonstrated that displacing the fingers of the seen image of the hand by only four centimetres relative to the participant’s true finger position reduced tactile performance as compared to when the seen and felt finger locations matched. Overall, it would seem that tactile processing is enhanced by vision of the site of touch when vision and proprioceptive references are aligned, but can be reduced when visual and proprioceptive information are spatially offset, even by a small amount.

In contrast, making asymmetrical movements during mirror visual feedback did not impair gap detection performance even though this condition should have induced a greater degree of sensorimotor incongruence insofar as the discrepancy between visual and proprioceptive-motor information was largest. We speculate that the greater spatial misalignment between the percept of the seen hand and that of the hand behind the mirror during asymmetrical movements could disrupt the integration of this information. The participants’ subjective ratings of the strength of the illusion provide indirect support for this suggestion in that the perceived strength of the illusion was significantly lower for the asymmetrical as compared to symmetrical movement condition. Alternatively, greater tactile sensitivity may have arisen during asymmetrical movements as compared to symmetrical movements with mirror visual feedback because the *novel* experience of ‘seeing’ the hand move in the opposite direction to the felt movement may have enhanced attention to the limb. These are necessarily post hoc explanations for our findings, but they are nonetheless consistent with the fact that sensitivity during the asymmetrical movement condition was not significantly different to sensitivity during the two board conditions.

Previous studies support the idea that illusions of body ownership may be sensitive to the degree of spatial congruence between the different sensory information that is used to evoke the illusion [43–45]; although spatial congruence is certainly not always necessary for multisensory integration [46]. The visuotactile integration that underlies the RHI is robust to offsets between the seen and felt hand of 10° [47] but is disrupted by spatial misalignments of 20° to 180° [47–49]. Furthermore, Kalckert and Ehrsson [50] investigated the effect of vertical distance between the real and rubber hands on the traditional RHI and a variant in which the illusion is induced through symmetrical movements of the real and rubber hands (the “moving” RHI). They found that proprioceptive drift was observed for the moving RHI when there was a relatively small spatial discrepancy, but not when there was a larger spatial discrepancy. In contrast, the traditional RHI elicited proprioceptive drift for both small and large spatial discrepancies, suggesting that spatial congruence may be more important for inducing the illusion of body ownership through sensorimotor integration than through crossmodal integration. In sum, these findings are consistent with the possibility that the large discrepancy between sensory input and motor intention prevents or reduces sensorimotor integration.

These results conflict with previous studies in which participants reported anomalous sensations during mirror visual feedback either more frequently or with equal frequency when making asymmetrical movements compared to when making symmetrical movements [13,16–18]. The main difference between the current experiment and previous studies is that we tested the effects of sensorimotor incongruence on responses to an evoked sensation–that is, to stimulation that originated from a source that is external to the body. It is possible that stimulation from a source that is external to the body provides a more robust signal that is less likely to be misinterpreted when there is a large discrepancy between sensory input and motor intention. In contrast, signals for visceral limb sensations may well be more vulnerable to misinterpretation, and may therefore arise–and indeed be more extreme—when there are large discrepancies between visual and proprioceptive-motor signals. Further studies will be required to determine whether the effects of sensorimotor incongruence on reported visceral limb sensations are different to those effects on responses to external stimulation.

Although we found reduced sensitivity (*d’*) during symmetrical movements with mirror visual feedback, there was no significant difference in response criterion (*c*) across the four conditions. In signal detection theory [24,51], sensitivity reflects the true separation between signal and noise, independent of response bias. In contrast, the criterion reflects the participants’ tendency to favor one response over another. The results of the present study therefore showed that sensitivity to the presence of the temporal gap was reduced, but participant’s inclination to report it remained unchanged. This suggests that the modulation in gap detection performance was related to changes in signal processing and not to changes in the decision-based component of the task. This result may likely reflect the fact that the gap length was set to the 50% detection threshold, as determined during the pre-test. We set the gap length to 50% detection threshold because in pilot testing that used longer gap lengths the participants tended to give perfect responses (i.e., responding ‘yes’ to all GAP trials and ‘no’ to all NO GAP trials). Note, however, that by choosing to limit the duration of the gap to 50% detection threshold, we might have closed the door to finding “true” anomalous sensations, as previously reported in the patient population. It may be that the previously reported tingling, foreignness, as well as the reported changes in the appreciation of weight or temperature, are just the result of adopting a decisional criterion, and may not reflect a change in sensitivity. Future studies are needed to investigate this decisional pathway.

Aside from the present study, only one other has examined the effect of sensorimotor incongruence on people’s responses to sensory stimulation originating from outside the body [14]. Wand and his colleagues [14] measured pressure pain thresholds in the resting forearms of healthy volunteers immediately after 40-second blocks of symmetrical or asymmetrical arm movements that were performed with direct vision of the arms or under mirror visual feedback. No differences in pain thresholds between the four conditions were found, thus suggesting that pain sensitivity was not altered by sensorimotor incongruence. This, along with the results of the present study, is consistent with McCabe and her colleagues’ [13] suggestion that anomalous sensations–including reduced tactile sensitivity–could be considered *warning signals* to alert the nervous system of potential errors in information processing, and that pain only results when the errors are particularly salient. It is possible that the threat presented by sensorimotor incongruence during mirror visual feedback is not sufficient to influence pain processing in healthy volunteers, but is enough to trigger other sensory abnormalities. Alternatively, the effects of sensorimotor incongruence on sensations in healthy volunteers may be limited to the exposure period itself, in which case they would have been undetected by Wand and his colleagues, who measured pain thresholds immediately after, rather than during, exposure.

The precise cognitive and neural mechanisms underlying the relationship between sensorimotor incongruence and abnormal sensations are unknown but may involve changes to the way in which signals for lower level sensory information are selected or filtered [5]. Sensorimotor incongruence has been associated with higher regional cerebral blood flow in the right dorsolateral prefrontal cortex [52] and reduced alpha-band activity in the posterior parietal cortex [53]. These changes may well reflect the increased monitoring and attentional demands involved in maintaining movement programs and integrating sensory and motor signals about the limb in the face of disparate information [54,55]. Changes to these monitoring processes could lead to the processing of incoming sensory inputs that would normally be suppressed–thus potentially leading to reports of tingling and numbness—or suppression of sensory inputs—leading to decreased tactile sensitivity.

Furthermore, those individuals who reported higher levels of discomfort during exposure to sensorimotor incongruence [53] showed decreased alpha band activity in the anterior cingulate cortex (ACC) and posterior cingulate cortex (PCC) compared to individuals who reported lower levels of discomfort. The ACC and PCC are associated with affective and cognitive components of pain [56,57], and pain-evoked brain responses in these areas are different in those with chronic pain conditions as compared to healthy controls [58–60]. This could be a mechanism through which sensorimotor incongruence evokes cognitive-affective responses, such as the reported feelings of foreignness.

In conclusion, distorted body representation is thought to contribute to several conditions of chronic pain and sensory disturbance [4,6,61–63]. The results of the present study provide partial support for the hypothesis that abnormal processing of evoked stimuli may arise due to incongruence between cortical signalling of motor intention and sensory input. Furthermore, the results reported here suggest that performing asymmetrical movements with mirror visual feedback may not necessarily evoke a higher degree of sensorimotor incongruence, as compared to performing symmetrical movements with mirror visual feedback. Developing an objective measure of the extent to which an individual experiences the mirror visual feedback illusion under different movement conditions would be important for any future work that aims to use this technique to induce sensorimotor incongruence.

## Supporting Information

S1 Table Individual participant data. (XLSX)
